# The Role of *n*-3 Long Chain Polyunsaturated Fatty Acids in Cardiovascular Disease Prevention, and Interactions with Statins

**DOI:** 10.3390/nu10060775

**Published:** 2018-06-15

**Authors:** Julia K. Bird, Philip C. Calder, Manfred Eggersdorfer

**Affiliations:** 1 DSM Nutritional Products, 4303 Kaiseraugst, Switzerland; manfred.eggersdorfer@dsm.com; 2 Human Development and Health Academic Unit, Faculty of Medicine, University of Southampton, Southampton SO16 6YD, UK; pcc@soton.ac.uk; 3 NIHR Southampton Biomedical Research Centre, University Hospital Southampton NHS Foundation Trust and University of Southampton, Southampton SO16 6YD, UK

**Keywords:** omega-3, cardiovascular disease, statins

## Abstract

Decreases in global cardiovascular disease (CVD) mortality and morbidity in recent decades can be partly attributed to cholesterol reduction through statin use. *n*-3 long chain polyunsaturated fatty acids are recommended by some authorities for primary and secondary CVD prevention, and for triglyceride reduction. The residual risk of CVD that remains after statin therapy may potentially be reduced by *n*-3 long chain polyunsaturated fatty acids. However, the effects of concomitant use of statins and *n*-3 long chain polyunsaturated fatty acids are not well understood. Pleiotropic effects of statins and *n*-3 long chain polyunsaturated fatty acids overlap. For example, cytochrome P450 enzymes that metabolize statins may affect *n*-3 long chain polyunsaturated fatty acid metabolism and vice versa. Clinical and mechanistic study results show both synergistic and antagonistic effects of statins and *n*-3 long chain polyunsaturated fatty acids when used in combination.

## 1. Introduction

Cardiovascular diseases (CVDs) are the leading cause of global mortality, accounting for 32% of the 56 million deaths in 2015 [1]. Despite declines in age-adjusted mortality rates of 22% over the last few decades, mostly in high income countries [2], mortality rates are expected to rise again due to shifts from infectious to chronic disease over the next decades [3]. CVDs contribute not only to mortality, but cause a considerable disease burden in healthy life years lost [4]. Reductions in cardiovascular risk factors such as smoking have contributed to half the drop in mortality, whereas the other half can be attributed to medical therapies that include the use of medications such as statins, niacin and fibrates in both primary and secondary prevention [5].

Statins are 3-hydroxy-3-methylglutaryl coenzyme A (HMG-CoA) reductase inhibitors and are currently considered standard of care in both primary and secondary prevention of CVD. Their main mode of action is to lower circulating cholesterol, mainly low-density lipoprotein (LDL) cholesterol, concentrations, thereby slowing or even reversing the development of atherosclerotic plaques [6]. A recent meta-analysis found that statins used as primary prevention reduced all-cause mortality by 14%, CVD by 25% and stroke events by 22% [7]. While lipid-lowering monotherapy has reduced overall cardiovascular mortality risk, even patients with a successful, aggressive reduction in LDL-cholesterol levels have a residual risk of myocardial infarction [8]. Risk factors other than elevated LDL-cholesterol have a marked influence on CVD incidence and mortality.

*n*-3 polyunsaturated fatty acids (PUFAs) have cardioprotective effects, particularly the two *n*-3 long chain (LC) PUFAs eicosapentaenoic acid (EPA) and docosahexaenoic acid (DHA) [9]. A landmark study comparing the diets and CVD rates of Greenland Inuit to the Danish population triggered the initial interest in the role of marine-derived *n*-3 PUFAs in CVD [10], and this was supported by epidemiological research associating fish consumption with a reduction in CVD mortality in other populations [11,12]. Various mechanisms have been proposed to explain the modest reductions in cardiovascular risk by *n*-3 LC PUFAs: these include preventing cardiac arrhythmias, lowering plasma triglycerides, reducing blood pressure, decreasing platelet aggregation, and reducing inflammation. Early randomized, controlled trials showed a reduction in risk of cardiovascular mortality after increasing consumption of fatty fish or *n*-3 LC PUFA dietary supplements [13,14,15]. However, some more recent intervention studies did not show significant effects of EPA and/or DHA supplementation [16].

One reason postulated for the lack of effect of *n*-3 LC PUFA supplements in the more recent secondary prevention studies conducted is the frequent use of statin therapy in study patients [17]. The mechanisms of action of *n*-3 LC PUFAs overlap with the pleiotropic effects of statins, such as improving endothelial function, and anti-thrombotic and antioxidant effects [18]. In addition, statins may affect PUFA concentrations and the production of eicosanoids through interactions with cytochrome P450 (CYP) enzymes. The use of statins may therefore interfere with the effects of *n*-3 LC PUFAs. The aim of this review is to explore the interrelationship between statins and *n*-3 LC PUFAs in the context of CVD.

## 2. Statins: Mode of Action

Statins decrease LDL-cholesterol levels and are classed as anti-dyslipidemic drugs. The mode of action common to all statins is the competitive inhibition of the activity of HMG-CoA reductase (HMGCR), the rate-limiting step in the endogenous production of cholesterol. The structure of statins mimics that of the cholesterol precursor HMG-CoA. Statins compete for binding sites on the HMGCR enzyme, slowing the rate of mevalonate production from HMG-CoA in the liver, which leads to a reduction in overall cholesterol production and also of other products downstream of mevalonate [19]. Statins consist of a HMG-like moiety with chemical side groups that affect their pharmacokinetics, lipophilicity, affinity to HMGCR, rate of entry into the liver and non-target cells, and associated side effects.

Pleiotropic effects of statins include improving endothelial function, inhibiting vascular inflammation, and the stabilization of atherosclerotic plaques [20]. Plaque stabilization and regression through statins may be caused by activation of peroxisome proliferator-activated receptors (PPARs). These effects may be related to the inhibition of isoprenoid synthesis by statins, which ultimately inhibits various intracellular signaling molecules.

Seven statins are approved for use in the United States and Europe for primary prevention of CVD in patients with hypercholesteremia or an elevated risk of CVD, and in secondary prevention in pre-existing CVD. Table 1 provides an overview of these statins and their classification. Of the marketed statins, fluvastatin, atorvastatin, rosuvastatin, and pitavastatin are synthetic molecules, while lovastatin, pravastatin and simvastatin are derived from compounds found in nature. Statin types have differing effects on LDL-cholesterol reduction and may be further classified as “weak” statins (pravastatin, simvastatin) and “strong” statins (rosuvastatin, pitavastatin, atorvastatin), loosely based on their ability to lower the concentration of LDL-cholesterol. Weak statins lower cholesterol by up to 25%, with strong statins achieving a greater reduction [21]. The degree of hydrophilicity is a further point of differentiation between statins as it affects their absorption, tissue selectivity, and metabolism by CYP enzymes in the liver [22]. The choice of statin is generally guided by the desired reduction in LDL-cholesterol concentration.

The effectiveness of statins for both cholesterol-lowering and prevention of cardiovascular mortality was recently confirmed once again with a systematic review and meta-analysis [27]. There was a dose-dependent reduction in CVD with LDL-cholesterol lowering. Across 27 trials, all-cause mortality was reduced by 10% per 1.0 mmol/L (approximately 40 mg/dL) LDL-cholesterol lowering, with LDL-cholesterol lowering largely reflecting a significant decrease in deaths due to coronary heart disease [27].

Even so, a residual CVD risk remains after statins are used, particularly in high-risk patients such as type II diabetics. Cardiovascular events occur even in patients that are adherent to intensive statin therapy and who achieve a large reduction in LDL-cholesterol to below 100 mg/dL [28]. CVD is multifactorial, and various risk factors that work through numerous mechanisms in the cardiovascular system affect the severity of disease risk. Statins mainly affect circulating concentrations of LDL-cholesterol, but other parameters of the lipid profile or markers of inflammation independently affect CVD progression and outcomes, such as HDL-cholesterol, LDL-cholesterol particle distribution, and elevated C-reactive protein and triglyceride concentrations [28,29]. The remaining risk of CVD outcomes in statin-treated patients is related to the independent effects of these risk factors and residual atherosclerosis. Combination therapy offers a means to treat other atherogenic components of the lipid profile, and also other risk factors. Common combinations include treatment with a fibrate to simultaneously lower triglyceride concentrations or with niacin to increase HDL-cholesterol [28].

## 3. Epidemiology of Statin Use

Since the introduction of lovastatin into clinical practice in 1987, statins have become one of the most widely prescribed classes of drugs in the world. The extensive use of statins has lowered LDL-cholesterol levels in the general population in high-income countries, and statins are considered to be one of the direct causes of the global reduction in cardiovascular events and mortality that has taken place in recent decades [30].

The largest markets for statins globally are the United States and Europe; however, the loss of patent exclusivity of the major brands since 2001 has opened the market for developing countries. In the United States, 93% of users of cholesterol-lowering prescription medication used a statin. 25% of adults aged 45 years and over used a statin in the period 2005–2008, equivalent to around 30 million adults [31], but usage varied widely depending on age and existence of CVD or diabetes [32]. Statin use varies greatly within Europe, with usage in countries such as Sweden, Ireland and the Netherlands four times greater than that in Austria or Italy [33]. The pattern of statin types prescribed also shows considerable variation among countries in the European Union [33]. In the United Kingdom, statins accounted for 94% of all prescriptions in the anti-dyslipidemic class in 2010: this corresponds to 55.1 million statin prescriptions dispensed in primary care. 72% of these prescriptions were for generic simvastatin [31]. Even so, treatment rates are still considered sub-optimal, and there is a considerable opportunity to reduce CVD through greater use in populations [30,32].

## 4. *n*-3 LC PUFAs: Mode of Action in CVD Prevention

The PUFAs linoleic acid (LA, *n*-6) and alpha-linolenic acid (ALA, *n*-3) are essential fatty acids: they are unable to be synthesized *de novo* by humans and must therefore be provided by the diet [34]. Intakes of LA and ALA less than 0.5% of energy are associated with deficiency symptoms, which include impaired barrier function and wound healing, failure to thrive, and can lead to poor neurological and visual development in infants [34]. Most vegetable oils are a good source of LA, and ALA may be obtained from selected vegetable oils including flaxseed, canola and soybean [34]. Dietary LA can function as a precursor for arachidonic acid (ARA). Likewise, ALA is a precursor for EPA and DHA, although conversion is rather poor in humans. Pre-formed EPA and DHA from fatty fish remains a more important dietary source of *n*-3 LC-PUFAs [35,36]. The *n*-3 LC PUFA DHA is regarded as conditionally essential for neonates for normal visual and cognitive development [34]. For adults, intakes of 250–2000 mg per day of EPA + DHA contribute to coronary heart disease prevention, and possibly to prevention of other chronic, degenerative diseases [34].

**Figure 1:**
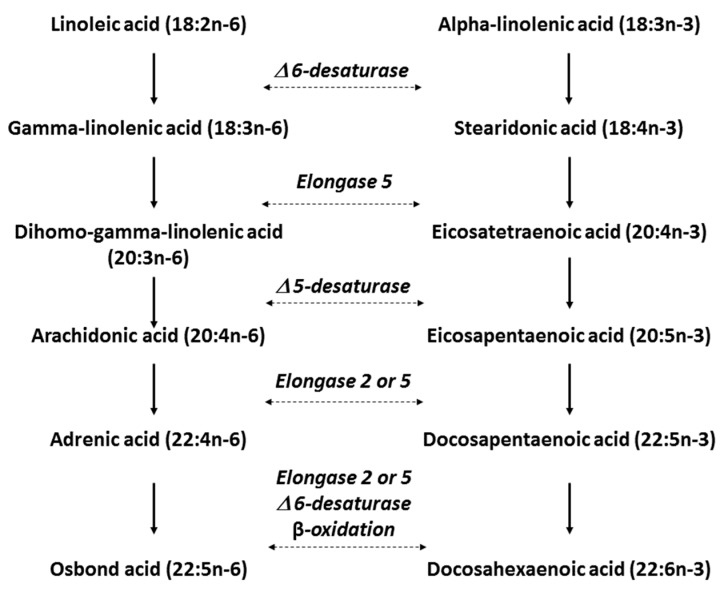
Pathway of metabolic interconversion of omega-6 and omega-3 polyunsaturated fatty acids. Abbreviation used: Δ, delta.

*n*-3 LC PUFAs are incorporated into triglycerides, phospholipids, and cholesteryl esters in plasma after absorption. There is a high correlation between EPA + DHA in erythrocytes, whole blood and plasma [37]. DHA is the most abundant *n*-3 fatty acid in cell membranes, being present in all organs, particularly in the cerebral cortex, the retina and in sperm [38]. EPA is also present in cells and tissues, albeit at considerably lower concentrations than DHA [36]. Both human plasma and tissues respond dose-dependently to supplementation with ALA, EPA and DHA [36]. Steady-state concentrations are reached after 1 month in plasma, and after 4–6 months in red blood cells; higher doses also lead to a faster response [36]. Interconversion from ALA to EPA and DHA is achieved in the liver via the sequential addition of 2-carbon units to the fatty acid backbone using elongation and desaturation enzymes until the chain length reaches 24 carbon units (Figure 1). The final step of conversion to DHA requires peroxisomal beta-oxidation. This last step is highly inefficient, particularly for men, with less than 1% of ALA intake ultimately converted to DHA [36]. Increasing doses of ALA will increase ALA and EPA concentrations in plasma but result in no discernable change in DHA concentrations [36]. The same enzyme system is also used for the elongation of *n*-6 PUFAs, therefore high background *n*-6 PUFA intakes reduce interconversion of *n*-3 PUFAs through competition (see Figure 1). Retroconversion of DHA to shorter chain *n*-3 PUFAs also occurs, albeit at a low rate of approximately 1.4% of a single dose [39]. Higher rates of retroconversion above 10% are suggested in individuals with high chronic intakes of DHA [40,41].

The ratio of EPA + DHA to total fatty acids is considered to be important as supplementation with EPA + DHA displaces ARA from plasma and tissues [36]. The saturation of the fatty acids and their chain length in the phospholipid bilayer of cell membranes affect its permeability, and physical state which in turn influences receptor function and the efficiency of signaling pathways [37]. The chain length of incorporated PUFAs affects membrane order; transmembrane protein activity is conferred by longer molecules with a greater number of double bonds. Of importance to CVD prevention, *n*-3 LC PUFAs reduce blood triglycerides, modulate the excitability of myocytes to reduce arrhythmias after damage to the heart, slow the progression of atherosclerosis, are mildly hypotensive, anti-inflammatory and promote endothelial relaxation, are anti-thrombotic, and are associated with a modest reduction in risk of cardiac death [17,34,42,43].

The effect of both EPA and DHA on lowering blood triglyceride concentrations is well established and is the basis for the use of ethyl esters of both molecules as a prescription medication for patients with hypertriglyceridemia [44]. The mechanisms of action are not completely understood; however, it is thought that a combination of reduced triglyceride synthesis and increased oxidation of triglycerides induced by *n*-3 LC PUFAs act to lower circulating triglyceride concentrations. *n*-3 LC PUFAs inhibit the hepatic synthesis and secretion of VLDL-triglycerides, mediated by decreases in transcription factors controlling the expression of enzymes involved in the assembly of triglycerides. Some studies link the down-regulation of sterol regulatory element-binding proteins (SREBPs), transcription factors required for the biosynthesis of both cholesterol and fatty acids, with reduced triglyceride synthesis in animal models seen following fish oil feeding. An increase in acyl-coenzyme A oxidase gene expression induced by PPAR-α may increase rates of peroxisomal β-oxidation of fatty acids. While both EPA and DHA lower triglycerides, head-to-head studies find that supplementation with DHA alone can raise LDL-cholesterol to a small extent but EPA does not [45]. The atherogenic potential of this increase in LDL-cholesterol may be mitigated by a shift in LDL particle size towards larger, more buoyant LDL particles after DHA supplementation [46,47]. On the other hand, DHA supplementation was associated with a modest increase in HDL-cholesterol, while increases due to EPA were comparatively minor [45].

The reduction in mortality found in some supplementation studies with *n*-3 LC PUFAs is attributed primarily to reductions in sudden cardiac deaths from decreased arrhythmogenesis [9,17,42]. Specifically, *n*-3 LC PUFAs are incorporated into the phospholipids of myocyte plasma membranes, where they have the ability to modulate cellular ion currents. EPA reduced myocyte excitability by increasing the time taken to return sodium channels in the membrane to their active state; however, this only occurs in cells that have become hyper-excitable due to damage such as ischemia [43]. Small to medium-sized intervention studies with *n*-3 LC PUFAs in secondary prevention support this mechanism by showing a reduction in both atrial fibrillation and ventricular arrhythmias in patients with frequent premature ventricular complexes or after coronary artery bypass surgery [42]. This may explain why a reduced risk of coronary heart disease with EPA + DHA from food or supplements was found in a recent meta-analysis, particularly in people with a higher risk of CVD [48]. *n*-3 LC PUFAs may therefore be most effective in reducing sudden cardiac death when cardiac tissue has already been injured.

A reduction in the risk of stroke is a clinically relevant outcome of the anti-thrombogenic and hypotensive effects of *n*-3 LC PUFAs [49,50], although results from some intervention studies have been indeterminate or only applicable to certain sub-groups [51,52,53]. Anti-thrombotic effects are thought to be primarily caused by the exchange of ARA with EPA in membrane phospholipids of blood platelets, causing favorable reductions in thrombogenicity due to enhanced production of non-aggregatory eicosanoids from EPA. The promotion of endothelial relaxation through stimulating nitric oxide synthesis in the endothelium has been demonstrated [54], but other effects on vascular reactivity independent of the endothelium are considered to be important contributors to the reduction in blood pressure found in intervention trials with *n*-3 LC PUFAs [55]. The slowing of atherosclerosis progression is related to the modulation of the expression and transcription of genes involved in the inflammatory response. Both EPA and DHA affect the nuclear factor-κB signal transduction pathway to reduce inflammation: EPA decreases the expression of tumor necrosis factor-α by impeding phosphorylation of nuclear factor-κB, while DHA reduces the ability of nuclear factor-κB to bind to DNA in an ischemia–reperfusion model [54]. It is likely that synergy between anti-inflammatory mechanisms, triglyceride lowering, improving membrane order, anti-thrombotic and anti-arrhythmic effects contributes to the overall reduction in CVD risk from *n*-3 LC PUFAs.

## 5. Use of *n*-3 LC PUFAs as Dietary Supplements in the General Population

Dietary supplements containing *n*-3 LC PUFAs are used widely in North America, Europe, and the Asia-Pacific region. The main sources of these products include fatty fish, krill, and fermentation-derived microalgal oils. Demand for *n*-3 LC PUFAs for human nutrition is projected to grow 4.1% annually on a volume basis over the coming decade [56]. An international survey of *n*-3 LC PUFA supplement users in ten countries (U.S., U.K., Germany, Italy, China, South Korea, Russia, Australia, Brazil, Mexico) found that usage varied from 14% of the adult population in Germany to 38% in Australia [57]. A high proportion of *n*-3 LC PUFA supplement use has also been found by other researchers [58]. In most countries, users started taking supplements due to advice from a physician. The main reasons given for taking supplements are for overall or cardiovascular health [57]. *n*-3 LC PUFA supplements are taken by 10% of the U.S. adult population aged 20 years or more, most commonly for heart health [59].

## 6. Interactions between LC PUFAs and Statins, and Effects on Dyslipidemia, CVD and Mortality

**Figure 2:**
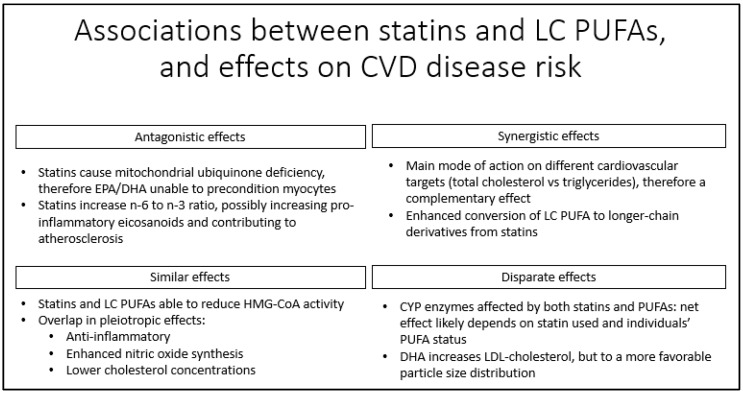
Overview of the interaction between statins and *n*-3 LC PUFAs on cardiovascular risk factor.

In addition to distinct effects on dyslipidemia, the pleiotropic effects of statins overlap with those of *n*-3 LC PUFAs. Similar mechanisms include enhancing endothelial nitric oxide synthesis, inhibiting the production of pro-inflammatory cytokines, and the lowering of LDL-cholesterol via repression in activity and mRNA expression of the HMG-CoA reductase enzyme [60,61]. Given these commonalities in actions, statins and *n*-3 LC PUFAs may interact, either competing with or complementing each other. Statins may also augment the metabolism of LC PUFAs and their metabolites [60]. These interactions are summarized in Figure 2.

### 6.1. Effects of Dietary Fatty Acids and Statin Co-Administration on Dyslipidemia

There is a well-established link between dietary fat type and the blood lipid profile. Dietary manipulation of fatty acid unsaturation affects both total circulating cholesterol concentration and various cholesterol fractions. For example, replacement of saturated fats in the diet with polyunsaturated and monounsaturated fats lowers total cholesterol and LDL-cholesterol concentrations [25,34]. The LDL-cholesterol lowering effect is thought to be due to the increased expression of LDL-cholesterol receptors in response to a higher concentration of unsaturated fatty acids. In contrast, saturated fatty acids maintain a lower expression of LDL-cholesterol receptors and thus LDL-cholesterol concentrations remain high [25], with the notable exception of LDL-cholesterol-lowering stearic acid [62].

Cholesterol levels may also be affected by EFA status through the modulation of HMG CoA reductase activity. In animal models, EFA deficiency increased the activity of HMG CoA reductase, possibly to help maintain barrier function and integrity when the supply of EFA is low [63]. Reversing deficiency normalized HMG CoA reductase activity [63]. Likewise, feeding studies in rodents show reduced HMG CoA reductase activity or expression after *n*-3 LC PUFA administration [64,65,66,67].

Dietary fatty acids can influence statin pharmacokinetics. When combined with simvastatin treatment, patients consuming a diet using olive oil as the primary culinary fat had a more favorable change in calculated risk of CVD, based on serum lipid and lipoprotein concentrations, than patients using sunflower oil. The higher concentrations of linoleic acid in sunflower oil compared to olive oil was postulated to cause a comparatively greater activation of cytochrome P450 enzymes, leading to a reduction in statin half-life, affecting its ability to lower cholesterol [25].

In clinical studies investigating the effect of various statins with *n*-3 LC PUFAs on cardiovascular risk factors, predominantly performed in patients with elevated cholesterol and triglycerides, combined treatment resulted in decreases in triglycerides, total cholesterol, and thrombotic potential compared to statin-only [25]. In general, concomitant therapy with statin medication and *n*-3 LC PUFAs is considered to be complementary, and alongside a reduction in both elevated triglycerides and LDL-cholesterol, there is a trend to lower LDL-cholesterol particle size and a more favorable lipoprotein distribution [68].

### 6.2. Effects of Statins on n-3 LC PUFA Concentrations

Statins and EFAs interact to modulate fatty acid synthesis and metabolism. In particular, statins have the ability to alter *n*-3 LC PUFA concentrations [69]. Statins have differential effects on the activities of the Δ6- and Δ5-desaturase enzymes, and studies indicate increases in activity (simvastatin, rosuvastatin and pitavastatin [70,71]) or decreases (atorvastatin [72]), and PPARs can be activated [20]. This can lead to changes in the relative proportions of longer chain PUFAs.

In vitro studies show that statins increase concentrations of ARA and other LC PUFAs, possibly because elongation activity is enhanced [73,74]. A dietary intervention study conducted in 120 hypercholesterolemic men found marked changes in the fatty acid profile after treatment with simvastatin, notably ARA, which suggested that there were changes in the activity of enzymes involved in the elongation and desaturation of fatty acids [71]. Another clinical trial in 57 men with coronary heart disease found that simvastatin treatment increased circulating concentrations of ARA, with no effect on *n*-3 LC PUFAs or saturated fatty acids [75].

A rat model was used to determine the effect of atorvastatin on *n*-3 PUFAs in plasma, blood, and erythrocyte membranes [72]. While *n*-3 LC PUFA concentrations remained the same or increased in plasma and erythrocyte membranes, there were significant reductions in liver *n*-3 LC PUFA concentrations as a result of atorvastatin treatment. These changes in *n*-3 LC PUFAs in the liver coincided with decreases in the mRNA expression of fatty acid desaturase (*FADS*) 1 and 2 genes, which encode Δ5-desaturase and Δ6-desaturase, respectively, and of *ELOVL5* gene, which encodes a key fatty acid elongation enzyme [76].

A study of 1723 Japanese cardiology patients showed that use of any statin increased circulating ARA and reduced circulating concentrations of DHA relative to ARA, without affecting EPA [77]. Differential effects were seen with simvastatin compared to rosuvastatin or pitavastatin. In 106 hypercholesterolemic adults and in in vitro experiments, simvastatin appeared to enhance the conversion of linoleic acid and EPA to ARA and DHA, respectively [69,78]. On the other hand, rosuvastatin or pitavastatin decreased serum DHA levels without affecting ARA or EPA, and thereby increased the ARA/DHA ratio in 46 dyslipidemic patients [70]. A further study in 46 coronary artery disease (CAD) patients found that atorvastatin, rosuvastatin or pitavastatin reduced EPA and DHA concentrations in serum, in proportion to reductions in LDL-cholesterol, while concentrations of ARA were unchanged [79]. There was a correlation between reduction in serum EPA + DHA and LDL-lowering, producing counteractive effects on risk factors for atherosclerosis. This has led some researchers to conclude that “weak” statins (simvastatin, pravastatin) increase the ARA/EPA ratio, while “strong” statins (atorvastatin, rosuvastatin or pitavastatin) increase the ARA/DHA ratio. Hydrophilic statins may require a higher dose to affect linoleic acid conversion than lipophilic statins [74]. In any case, statin use in general appears to favor *n*-6 over *n*-3 LC PUFA content [77], which may result in a net increase in inflammation and thrombogenesis [80].

### 6.3. Interactions between Statins and n-3 LC PUFAs on Mitochondrial Function

There may be a counteracting effect of statins and *n*-3 LC PUFAs on mitochondrial function [80]. Myocardial mitochondria provide energy for ischemic pre-conditioning in cardiomyocytes prior to myocardial infarction, which may reduce the size of the infarction, reduce post-ischemic arrhythmias and result in better patient survival. Dietary *n*-3 LC PUFAs are able to induce a chronic state of cardiac preconditioning, associated with increases in *n*-3 LC PUFA accumulation in plasma as well as cardiac mitochondria [80]. On the other hand, a known side-effect of statin usage is muscle pain and weakness, linked to disrupted mitochondria in muscles [22]. Endogenous production of ubiquinone, used primarily to generate energy in mitochondria, is decreased by statin administration, as its biosynthesis requires the HMG-CoA reductase enzyme [81]. Therefore, in the presence of statins, *n*-3 LC PUFAs may not be able to precondition cardiomyocytes due to a reduction in mitochondrial function arising from intrinsic ubiquinone deficiency.

### 6.4. Inhibition of CYP Enzymes by Statins and Effects on Eicosanoid Production

An important biological function of LC PUFAs is the production of eicosanoids, lipid mediators with cardioprotective, vasodilatory, inflammatory and allergic properties [82]. ARA is considered the traditional precursor of eicosanoids; however the CYP enzymes responsible for metabolizing ARA have broad substrate specificities and accept most *n*-3 and *n*-6 LC PUFAs [82]. Increasing the availability of EPA and DHA to the CYP enzymes shifts eicosanoid production to EPA- and DHA-derived metabolites, possibly having a favorable effect on CVD risk [83]. However, statins can inhibit or induce the activity of particular CYP enzymes [22,24,84], and thus the production of CYP-derived eicosanoids. For example, fluvastatin is both a substrate for, and potent inhibitor of, CYP2C9 [84]. CYP2C9 is found in human cardiovascular tissue [85], where it catalyzes the conversion of ARA, EPA and DHA to epoxyeicosatrienoic acids (EETs), epoxyeicosatetraenoic acids (EpETE) and epoxydocosapentaenoic acids (EpDPE), respectively [86]. EpETE and EpDPE show in vitro anti-inflammatory and cardioprotective properties [82]. Use of fluvastatin may reduce the overall production of PUFA-derived eicosanoids, with differential effects on CVD risk depending on the ultimate shift in PUFA-derived eicosanoids that occurs. When underlying *n*-3 LC PUFA concentrations are high, statins may lower the effectiveness of EPA or DHA in reducing CVD risk by inhibiting the production of EpETEs and EpDPE. On the other hand, when *n*-6 LC PUFA concentrations are high, a reduction in the production of ARA-derived inflammatory metabolites through CYP2C9 inhibition by certain statins may be beneficial. As a complicating factor, different statins affect different CYP enzymes, and some have no effect. In populations using a range of different statins, disparate effects on eicosanoid production may increase variability in the effect of PUFA on cardiovascular risk. Clearly, further work is needed in this area.

### 6.5. Effects of Statins and n-3 LC PUFAs on Clinical and Mechanistic Endpoints

The effect of *n*-3 LC PUFAs and statins on various clinically relevant endpoints has been addressed in several observational and interventional studies. Clinical study results are summarized in Table 2. Three large studies contrasted the effects of *n*-3 LC PUFAs in statin users compared to non-users. The Southern Cohort Community study is a prospective cohort investigation into risk factors for chronic disease in the Southwestern USA [87]. Among 69,559 participants, there was a statistically significant reduction in all-cause mortality across quintiles of increasing *n*-3 fatty acid intakes in non-statin users only; there was no effect in statin users. The Alpha Omega clinical trial in the Netherlands tested the effect of margarines that provided 4153 participants with a randomly-selected dose of 400 mg EPA + DHA, 2 g ALA, a combination, or placebo on major cardiovascular events [88]. There was no effect of the fatty acid type on risk of composite cardiovascular endpoints in statin users. In non-users, there were non-significant decreases in cardiovascular events in all treatment groups compared to placebo, and the reduction bordered on significance in the adjusted model for the combined EPA + DHA + ALA group (*p* = 0.051). On the other hand, in a retrospective cohort study conducted in 11,269 survivors of acute myocardial infarction from five Italian Local Health Units, a significant reduction in recurrent myocardial infarction was only seen in users of concurrent *n*-3 LC PUFA supplements and statins [89]. Statin use did not affect all-cause mortality in this study, however; there was a significant reduction in both statin users (HR 0.52 [0.40–0.68]) and statin non-users (HR 0.39 [0.20–0.75]) who were taking the *n*-3 LC PUFA supplements.

Further clinical studies have investigated the effect of combined treatment with *n*-3 LC PUFAs and statins compared to treatment with statins alone on cardiovascular events. In a large retrospective cohort study conducted across Italy, combined treatment with a statin and *n*-3 LC PUFAs (non-specified) after acute myocardial infarction was associated with improved survival in both crude and adjusted estimates for major outcomes compared to treatment with only a statin [90]. The large randomized controlled study JELIS, conducted in Japanese patients with hypercholesterolemia, tested the effect of adding 1800 mg EPA daily to existing statin treatment (10 mg pravastatin or 5 mg simvastatin) [91]. Compared to statin treatment alone, patients randomized to additional EPA had a lower incidence of major coronary events. In a recent open-label, randomized, controlled trial, acute coronary syndrome patients who were randomized to EPA + statin (1800 mg EPA ethyl ester and 2 mg pitavastatin daily) had approximately half the rate of a composite endpoint consisting of cardiovascular death, MI, stroke, or coronary revascularization at 1 year, compared to patients randomized to the statin treatment alone [94].

Several studies conducted in patients taking statins show that *n*-3 LC PUFA use in addition to statins affects CVD mechanisms. In 20 adults with familial hypercholesterolemia on stable statin therapy, an 8-week intervention with 4 g per day *n*-3 LC PUFAs (46% EPA and 38% DHA) resulted in improved arterial elasticity, and reduced blood pressure, apolipoprotein B and triglycerides, compared to statin therapy alone. In this risk group for premature CVD, *n*-3 LC PUFAs improved several independent predictors of CVD in addition to the normalization of cholesterol from statins [96]. In a small study that explored the mechanisms of cardiovascular risk factors in 200 patients treated with pitavastatin both alone and combined with 1800 mg EPA, there was a significantly higher rate of plaque regression in the combination group compared to pitavastatin alone [93]. In a similar recent RCT of patients with stable coronary artery disease on statin therapy, adherent patients randomized to 3.4 g EPA + DHA per day had less progression of fibrous coronary plaques, compared to statin therapy alone [95]. Both these studies show that atherosclerotic plaques regressed when a combination of *n*-3 LC PUFAs and statins were used.

Combination therapy of *n*-3 LC PUFAs and statins has shown some potential for patients who show poor tolerability or a lack of response to statin treatment. For example, in patients with moderate hypertriglyceridemia despite statin treatment, a combination of low dose rosuvastatin (10 mg) and *n*-3 LC PUFAs (2 g EPA + DHA) reduced total cholesterol and triglycerides compared to baseline [97]. While this reduction was not as great as for high dose rosuvastatin (40 mg), it showed clinical benefit and may be an option for patients with poor tolerability for high dose rosuvastatin. Another small clinical trial investigating the use of *n*-3 LC PUFAs and phytochemicals as complementary therapy to reduce statin dose used a personalized approach. In the first phase of the study, patients responding to treatment with 1.7 g *n*-3 LC PUFAs were identified and assigned to receive a halved statin dose in the second phase of the study. Despite a marked reduction in dose, there were no significant changes in the lipid profile in responders taking the combination therapy [98].

The research involving clinical and mechanistic endpoints is equivocal: some studies show that the combination of *n*-3 LC PUFAs and statins is beneficial, while others show no difference in outcomes, and yet others find that *n*-3 LC PUFAs only affect outcomes in statin non-users. Yet, in the period in which statin use has transitioned to becoming a first-line medication for reducing mortality derived from hypercholesterolemia, the results of supplementation studies with *n*-3 LC PUFAs have changed from showing a significant reduction in all-cause mortality increasingly to a null-effect [52], although a modest reduction in cardiac death or coronary heart disease risk has been found in two recent meta-analyses [17,48]. The disparate results of meta-analyses arise at least partly from variations in inclusion criteria, with Maki et al. reporting that their inclusion of smaller trials contributed to the robustness of the meta-analysis results [17]. Differences in *n*-3 LC PUFA doses used and the formulation or dosage form may be important: lower doses are less effective in raising circulating fatty acid concentrations, and the bioavailability from a food matrix may be subject to greater variability than from a dietary supplement. In addition, the distinct effects of DHA compared to EPA cannot be elucidated due to a paucity of comparative studies [55]. Furthermore, the effect size of *n*-3 LC PUFAs on mortality may have become smaller against a background of increasing statin use in the general population, leading to a higher likelihood of type I error. Overlapping or even counteractive effects of combined treatment with statins and *n*-3 LC PUFAs may be confounding outcomes of clinical trials.

## 7. Conclusions

Both statins and *n*-3 LC PUFAs are recommended for CVD prevention. While each treatment has a distinct mode of action, pleiotropic effects of the two overlap. In addition, statins and *n*-3 LC PUFAs interact, potentially affecting net cardiovascular risk (Figure 2). Statins may cause a mitochondrial ubiquinone deficiency, which blocks the ability of *n*-3 LC PUFAs to precondition myocytes, reducing their effectiveness in reducing cardiac arrhythmias. Statins appear to increase concentrations of LC PUFAs: when LA intakes are high, this could lead to a rise in concentrations of pro-inflammatory eicosanoids from ARA. The main effect of statins is to block the activity of HMG-CoA reductase; however *n*-3 LC PUFAs are also capable of HMG-CoA reductase inhibition, albeit less effectively, resulting in a smaller effect size for the combination. Both competition for, and activation of, CYP enzymes could be a further confounding factor in the metabolism of statins and the production of eicosanoids from *n*-3 LC PUFAs, but this may depend on the type of statin used. Post hoc analyses of clinical studies have yielded mixed results, with some results indicating that *n*-3 LC PUFA supplementation is only beneficial in statin non-users and others showing combined use of *n*-3 LC PUFA and statins is beneficial. Prospective intervention studies that stratify for statin use are warranted to explore the interaction further.

## Figures and Tables

**Table 1:** Statin classifications.

Statin Name	Origin [23]	Structure [23]	Lipophilicity [23]	Generation [23]	CYP Metabolism [22,24,25]
Fluvastatin	Synthetic	Fluorophenyl group	Lipophilic	I	CYP2C9
Atorvastatin	Synthetic	Fluorophenyl group	Lipophilic	II	CYP3A4
Rosuvastatin	Synthetic	Fluorophenyl group	Lipophobic	III	CYP2C9
Pitavastatin	Synthetic	Fluorophenyl group	Lipophilic	II	Marginal [26]
Lovastatin	Fungal	Butyryl group	Lipophilic	I	CYP3A4
Pravastatin	Fungal	Butyryl group	Lipophobic	I	CYP2C9
Simvastatin	Fungal	Butyryl group	Lipophilic	II	CYP3A4

Cytochrome P450 (CYP).

**Table 2:** Clinical studies with a combination of *n*-3 LC PUFAs and statins.

Study Name (*n*)	Study Type/Treatments	Main Results	Reference
Southern cohort community study (*n* = 69,559)	Prospective cohort study	Modest inverse associations between *n*-3 PUFA and *n*-6 PUFA intake with mortality among non-statin users but not among statin users.	[87]
(*n* = 14,704)	Retrospective cohort study	As compared with statins alone, combined treatment with statins and *n*-3 LC PUFAs was associated with an adjusted higher survival rate, survival free of atrial fibrillation and survival free of new heart failure development, but not with re-infarction.	[90]
JELIS (*n* = 18,645)	RCT Treatment: 1800 mg EPA + statin Control: statin alone	The incidence of MCE was significantly lower in the EPA group. Compared to patients with normal serum TG and HDL-C levels, those with abnormal had significantly higher CAD hazard ratio. In this higher risk group, EPA treatment suppressed the risk of CAD by 53%.	[91,92]
Alpha Omega (*n* = 4153)	Post hoc analysis of RCT Treatments: 400 mg EPA + DHA 2 g ALA Both Control: Placebo margarine	In statin users, *n*-3 fatty acids did not reduce cardiovascular events. In statin non-users, only 9% of those who received EPA-DHA plus ALA experienced an event compared with 18% in the placebo group.	[88]
CHERRY (*n* = 193)	RCT Treatment: 1,800 mg EPA + 4 mg pitavastatin Control: Pitavastatin (Pitavastatin) 4 mg	The prevalence rate of plaque regression was significantly higher in Pitavastatin/EPA group than in Pitavastatin group (50% vs. 24%, *p* < 0.001).	[93]
Kagawa hospital study (*n* = 241)	Prospective, open-label, randomized trial Treatment: 1,800 mg EPA + 2 mg Pitavastatin Control: 2 mg Pitavastatin	Significant reduction in composite endpoint of cardiovascular death, MI, stroke, or coronary revascularization at 1 year: 9.2% in the EPA group and 20.2% in the control group (absolute risk reduction, 11.0%; HR, 0.42; 95% CI, 0.21–087; *p* = 0.02), in acute coronary syndrome patients.	[94]
(*n* = 11,269)	Retrospective cohort study	*n*-3 LC PUFA supplement users had a reduced risk of all-cause mortality (HR 0.76 [0.59 to 0.97]). Statin use did not affect all-cause mortality reduction, however a reduction in recurrent myocardial infarction was only seen in statin users.	[89]
(*n* = 77,776)	Meta-regression	Lower control group statin use and higher DHA/EPA ratio was associated with higher reduction in total mortality.	[18]
HEARTS (*n* = 285)	RCT in patients with stable statin therapy Treatment: 1860 mg EPA + 1500 mg DHA Control: Placebo	EPA + DHA in addition to low-dose statin treatment prevented progression of atherosclerotic plaques, compared to low-dose statin treatment alone.	[95]

Abbreviations: coronary artery disease (CAD), alpha-linolenic acid (ALA, *n*-3), randomized clinical trial (RCT), major coronary event (MCE), triglycerides (TG), coronary artery disease (CAD), myocardial infarction (MI), eicosapentaenoic acid (EPA), hazard ratio (HR), docosahexaenoic acid (DHA).
